# Trabectedin as second-line treatment in metastatic myxoid liposarcoma: a case report

**DOI:** 10.1186/1752-1947-6-424

**Published:** 2012-12-19

**Authors:** Irene Zarcos Pedrinaci, José Miguel Jurado, Josefa Carrillo, Mercedes Caba Molina

**Affiliations:** 1 Medical Oncology Department, Hospital Clínico San Cecilio de Granada, Granada, Spain; 2 Pathology Department, Hospital Clínico San Cecilio de Granada, Granada, C/Dr Olóriz 16, Granada, 18012, Spain

## Abstract

**Introduction:**

Soft tissue sarcomas are heterogeneous tumors that are difficult to treat. Up to 50 percent of patients develop metastatic disease and require systemic chemotherapy. Ifosfamide and doxorubicin are the two most active agents.

**Case presentation:**

A 33-year-old Caucasian woman presented to our facility with a metastatic myxoid liposarcoma. Our patient was initially treated with surgery and radiation therapy, but experienced three recurrences during a six-year period, the first and the last occurring while our patient was pregnant. The first recurrence, which occurred two years after diagnosis and was localized in the left cervical and right axillary region, was treated with surgery followed by chemotherapy. Molecular analysis of this tumor showed a t(12,16) + translocation resulting in a FUS-DDIT3 or EWSR1-DDIT3 fusion. Three years later our patient experienced a second recurrence in the left supraclavicular fossa, upper thoracic and anterior mediastinum, which was treated with surgery alone. Eight months later, during the second pregnancy, our patient experienced a third recurrence as a large cervical mass that was treated, upon pregnancy, with trabectedin (1.5mg/m^2^/24-hour continuous infusion) for a total of 12 cycles. At that time a computed tomography scan showed long-term partial response with excellent treatment tolerability.

**Conclusions:**

This case report illustrates the potential therapeutic activity of trabectedin in patients with myxoid liposarcoma.

## Introduction

Soft tissue sarcomas (STS) are heterogeneous tumors that are difficult to treat. Up to 50 percent of patients develop metastatic disease and require systemic chemotherapy. Ifosfamide and doxorubicin are the two most active agents (response rates: 10 to 25 percent) [1]. Other alternative drugs have also been tested, with response rates less than 10 percent. Recently, trabectedin, which binds to the deoxyribonucleic acid (DNA) minor groove, has demonstrated significant activity in phase II studies in patients with STS, particularly those with leiomyosarcomas and liposarcomas (LPS), leading to its approval for this indication in the European Union [2]. Myxoid liposarcoma (ML) accounts for one-third of liposarcomas and represents a morphologic continuum encompassing myxoid and myxoid/round cell variants, the latter characterized by an extent of round cell components greater than five percent [3]. ML is associated with a chromosomal translocation resulting in FUS-CHOP or EWS-CHOP fusion proteins. The high response rates achieved with trabectedin in ML suggest a selective mechanism of action in this translocation-related sarcoma, representing a potential target for the development of more effective therapies. Additionally, early radiological changes in ML tumor tissue often precede tumor shrinkage, which may be delayed. Therefore, it seems important to integrate both density and size in the response evaluation [4].

Here, we present the case of a patient with metastatic LPS who achieved a prolonged partial response (PR) with trabectedin.

## Case presentation

**Figure 1:**
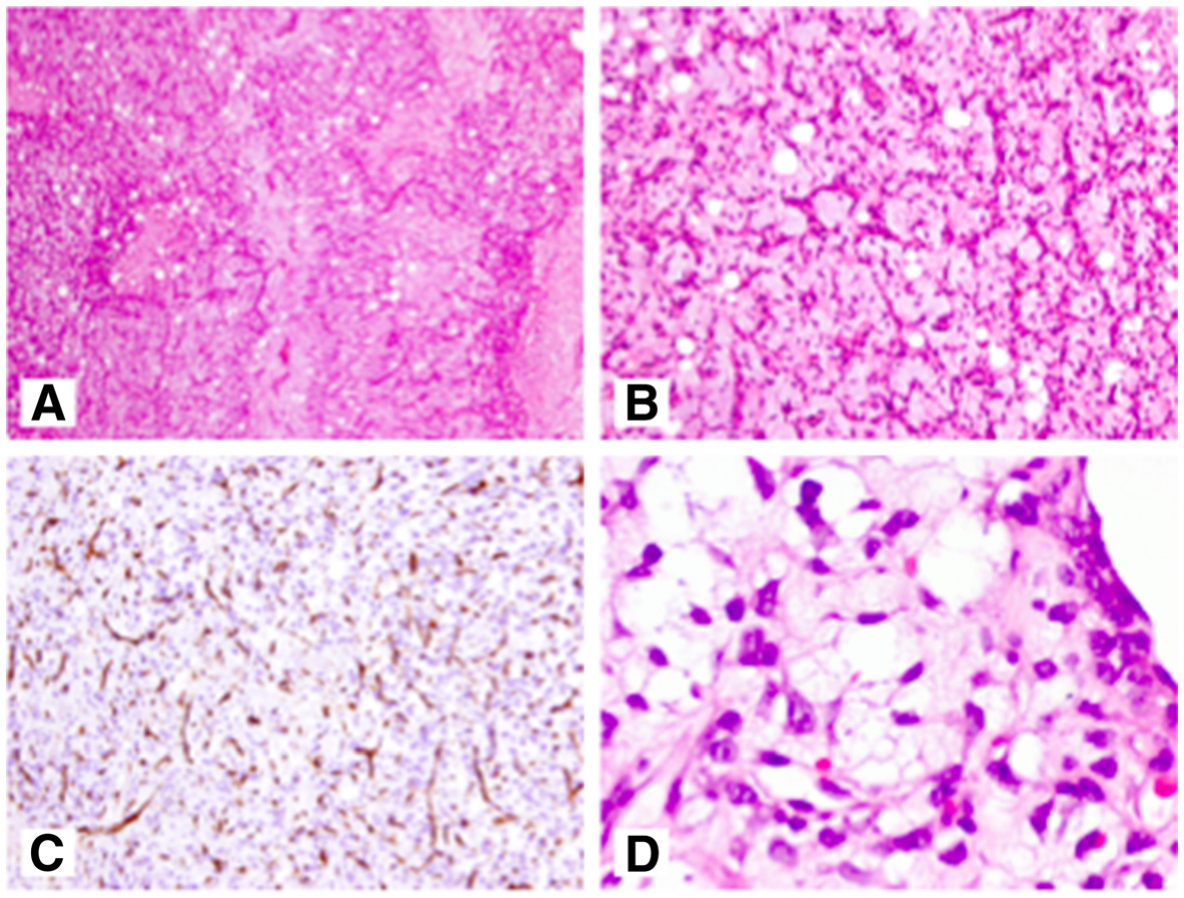
**Histology of myxoid liposarcoma. **Low magnification image of myxoid liposarcoma showing a variable number of adipocytes and a prominent capillary pattern ((**A**), hematoxylin and eosin stain, original magnification ×50); myxoid liposarcoma with relatively low cellularity showing uniform oval tumor cells and prominent, branching capillary pattern with variable numbers of non-atypical adipocytes ((**B**), hematoxylin and eosin stain, original magnification ×100); branching capillary pattern staining with CD34 ((**C**), original magnification ×100); and presence of some multi-vacuolated lipoblasts ((**D**), hematoxylin and eosin stain, original magnification ×600).

**Figure 2:**
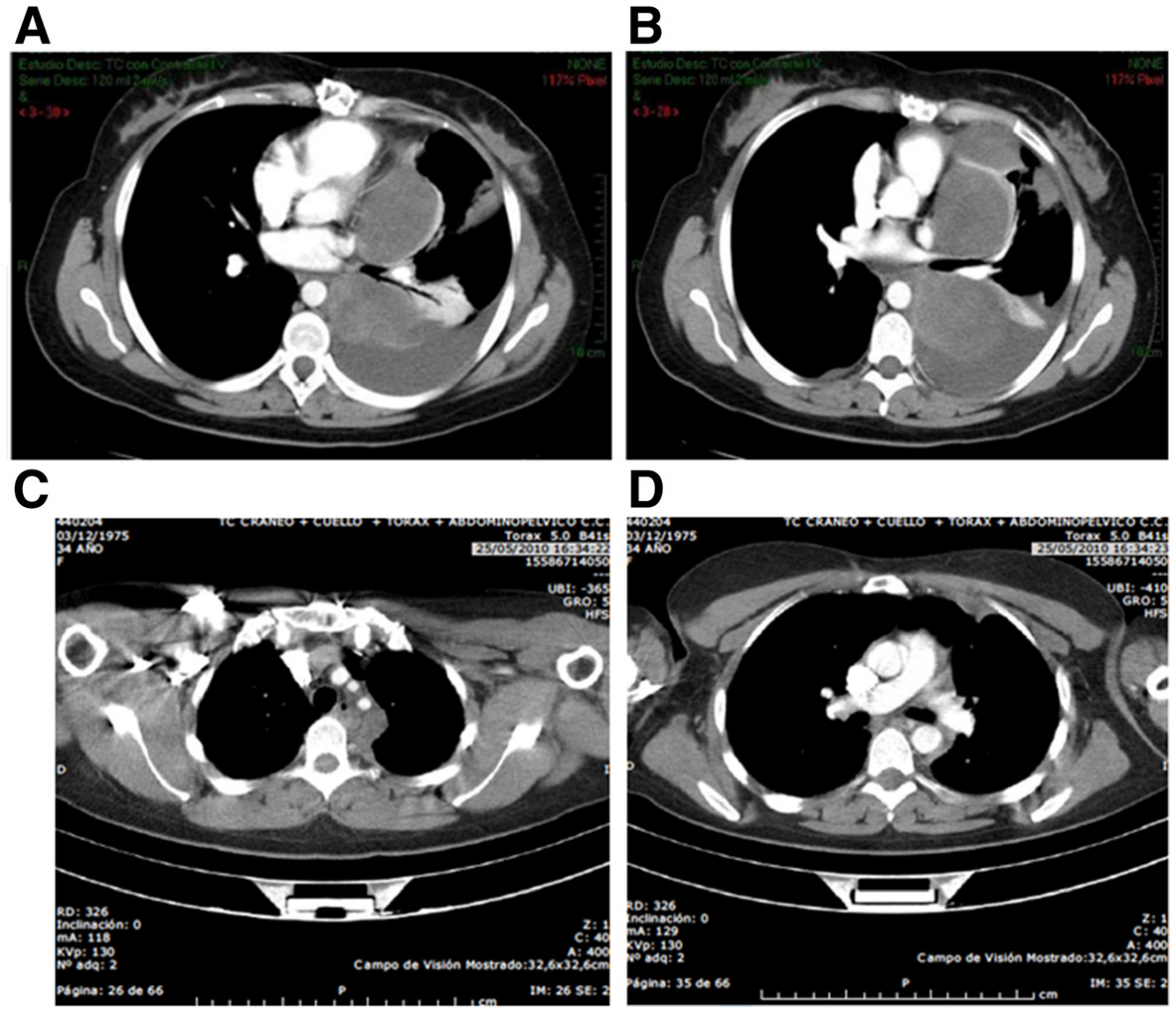
Computed tomography scan of the thoracic region, before (A,B) and after (C,D) treatment with trabectedin (12 cycles).

A 33-year-old Caucasian woman was diagnosed in 2006 with a localized myxoid LPS of the right thigh that was treated with compartmental resection of the right quadriceps, with histopathological results showing a 10.7cm low-grade ML (well differentiated, mitotic activity: <10 and <50 percent of necrosis), with infiltration of margins that required extension surgery up to the periosteum, without the presence of residual tumor in the margins. Subsequently, she received adjuvant treatment with external radiotherapy to a dose of 70Gy. Two years later, while pregnant, she noted a mass in her left cervical and right axillary region, which proved to be a metastatic low-grade LPS (Figure 1). Molecular analysis of this tumor showed a t(12,16) + translocation resulting in a FUS-DDIT3 or EWSR1-DDIT3 fusion. Our patient underwent negative margin resection followed by adjuvant chemotherapy with ifosfamide and epirubicin for six months. Three years later, the disease recurred with lesions in the left supraclavicular fossa (6.5×3.5cm) and upper thoracic and anterior mediastinum (4.5×5.0cm). The lesions were resected with no further treatment. Eight months later, during her second pregnancy, our patient noted a mass in her left cervical area. She delayed consultation for a few months until the baby was born and eventually presented with a large cervical mass that compressed the supra-aortic and carotid vessels and extended caudally to occupy the upper portion of the right hemi-thorax. There was also a second mass in the left pleural space; tumor size was 9×5cm (Figure 2A,B).

Our patient was given trabectedin (1.5mg/m^2^/24-hour continuous infusion) and after four cycles a significant clinical improvement in her dyspnea and pain was noted. A magnetic resonance imaging (MRI) scan showed a 50 percent reduction in the left supraclavicular mass, improvement in the chest lesion and resolution of the pleural effusion. This was considered stable disease by RECIST (Response Evaluation Criteria In Solid Tumors) criteria and a PR according to the criteria of Choi *et al.*[5]. She continued treatment for four additional cycles with complete response in the neck mass and significant reduction in the mediastinal and pleural lesions. A computed tomography scan after 12 cycles demonstrated a long-term PR according to RECIST criteria (Figure 2C,D). Our patient remained asymptomatic with good treatment tolerability (only two episodes of grade 2 and 3 neutropenia in cycles 3 and 4, respectively).

## Discussion

ML accounts for one-third of liposarcomas and represents a morphologic continuum encompassing myxoid and myxoid/round cell variants, the latter characterized by an extent of round cell components greater than five percent [6]. The molecular hallmark of ML is the presence of FUS-CHOP fusion proteins, with surgery and radiochemotherapy the standard therapy for localized ML. Surgery must include a wide excision with negative margins (R0). While 1cm has been selected as a cut-off point in some studies, it is important to realize that the margin can be minimal in the case of resistant anatomical barriers, such as muscular fasciae, periosteum and perineurium. Wide excision followed by radiation therapy is standard treatment in high-grade, deep lesions, >5cm. In the case of low-grade, deep, >5cm soft tissue sarcoma, radiation therapy should be discussed in a multidisciplinary fashion, taking into account the anatomical site and the related expected sequelae versus the histological aggressiveness. Overall, radiation therapy has been shown to improve local control, but not overall survival. Radiation therapy should be administered post-operatively, with the best technique available, at a dose of 50 to 60Gy, with fractions of 1.8 to 2Gy, possibly with boosts up to 66Gy depending on presentation and quality of surgery. Alternatively, radiotherapy may be carried out pre-operatively, normally using a dose of 50Gy. Intra-operative radiation therapy (IORT) and brachytherapy are also options for selected cases [3]. In our patient’s case, adjuvant treatment with external radiotherapy was considered even though she had a low-grade LPS because of its size (10cm) and because adequate margins could not be achieved. Our patient did not receive adjuvant chemotherapy. The results of some studies have shown that adjuvant chemotherapy might improve, or at least delay, distant and local recurrence in high-risk patients. However, a final demonstration of efficacy is lacking. It is also unknown whether adjuvant chemotherapy may be especially beneficial in specific subgroups. Therefore, adjuvant chemotherapy is not a standard treatment in adult-type soft tissue sarcomas. Clinical practice guidelines propose adjuvant chemotherapy as an option for high-risk individual patients, which includes those having a >G1, deep, >5cm tumor [6].

Our patient experienced three recurrences during a six-year period; two of them were managed with complete excision and the first was followed by adjuvant chemotherapy with ifosfamide and epirubicin for six months. Clinical guidelines recommend treating extrapulmonary disease recurrence with chemotherapy as standard. However, in selected cases, surgery of potentially resectable metastases may be offered as an option following a multidisciplinary evaluation, taking into consideration the site(s) and the natural history of the disease in the individual patient. Standard chemotherapy is based on anthracyclines as first-line treatment [4,6].

Only a few cases of recurrent LPS have been reported during pregnancy to date, and no solid evidence of an increased incidence or a worse outcome has been demonstrated. Trabectedin has shown activity in patients with STS with a response rate of eight percent and a 47 percent one-year survival. In a phase II study focused on patients with leiomyosarcomas and LPS, the agent demonstrated a 14-month median survival that is superior to the expected 12 months seen with historical controls [7]. One issue in assessing the activity of new agents in STS is the criteria for response. Tumor size may increase or remain stable in responding patients because of necrosis, bleeding and myxoid degeneration. For this reason, new criteria integrating tumor size and tumor density have been proposed. Molecular analysis of this tumor showed a t(12,16) + translocation resulting in a FUS-DDIT3 or EWSR1-DDIT3 fusion that has been related in pre-clinical studies with the activity of trabectedin in myxoid LPS [4].

## Conclusions

This case report highlights the activity of trabectedin on myxoid LPS with (12,16) + translocation resulting in a FUS-DDIT3 or EWSR1-DDIT3 fusion after failure of local and post-operative chemotherapy.

## Consent

Written informed consent was obtained from the patient for publication of this case report and any accompanying images. A copy of the written consent is available for review by the Editor-in-Chief of this journal.

## Competing interests

The authors declare that they have no competing interests.

## Authors’ contributions

All authors made substantial contributions to all of the following: (1) the acquisition of data; (2) drafting of the article and (3) final approval of the version to be submitted. All authors read and approved the final manuscript.
